# Sickness absence and disability pension among women with breast cancer: a population-based cohort study from Sweden

**DOI:** 10.1186/s12889-021-10703-1

**Published:** 2021-04-09

**Authors:** Pia K. Kvillemo, Lingjing Chen, Matteo Bottai, Paolo Frumento, Gino Almondo, Ellenor Mittendorfer-Rutz, Emilie Friberg, Kristina A. E. Alexanderson

**Affiliations:** 1 Division of Insurance Medicine, Department of Clinical Neuroscience, Karolinska Institutet, SE-171 77 Stockholm, Sweden; 2 Division of Biostatistics, Institute of Environmental Medicine, Karolinska Institutet, Stockholm, Sweden; 3 Department of Political Sciences, University of Pisa, Pisa, Italy

**Keywords:** Sick leave, Breast cancer, Diagnosis specific, Cohort-study, Predictive model, Real-world data, Insurance medicine

## Abstract

**Background:**

Women’s return to work after diagnosis of breast cancer (BC) is becoming more prevalent. However, register-based national investigation on sickness absence (SA) and disability pension (DP) in BC women is lacking. The aim of the study was to explore SA and DP before and after a first BC diagnosis and the possibility to predict new cancer-related SA by using disease-related and sociodemographic factors.

**Methods:**

A longitudinal register study of the 3536 women in Sweden aged 19–64 with a first BC diagnosis in 2010 was conducted by linkage of five nationwide registers. Particularly, detailed information on SA and DP was obtained from the National Social Insurance Agency. Descriptive statistics on SA and DP 2 years before through 3 years after the BC diagnosis were performed. The risk of having a new SA spell due to BC or BC-related diagnoses was modeled using logistic regression.

**Results:**

The proportion of women with SA increased during the year following the BC diagnosis date and declined over the next 2 years to proportions before diagnosis. At the time of BC diagnosis, half of the women began a new SA spell > 14 days with cancer, cancer-related, or mental diagnosis. Disease-related and sociodemographic factors including occupational sector, living area, age, cancer stage, educational level, and number of previous SA days showed statistical significance (*p* < 0.05) in predicting a new SA around BC diagnosis. By using these factors, it was possible to correctly predict 67% of the new SA spell.

**Conclusions:**

SA among women with BC was elevated mainly in the first year after diagnosis. New SA following BC diagnosis can accurately be predicted.

## Introduction

Breast cancer (BC) is a major health problem with 1.67 million new cases worldwide annually [1]. Due to early detection and better treatments, mortality has decreased, hence more knowledge is needed on potential adverse long-term social consequences of BC for the growing number of survivors [2–4]. About half of the women diagnosed with BC are of working age, [5, 6] thus, BC might imply sickness absence (SA) or even disability pension (DP) for many of them due to effects of BC and/or BC treatments. Studies indicate that many women with BC value paid work highly and want to continue working after diagnosis or return to work (RTW) as soon as possible [7–10], and more knowledge is needed on patterns of SA and DP in order to get the knowledge base for interventions to facilitate part- or full-time (return to) work.

Studies of SA, DP, and RTW among women with BC indicate that the majority of RTW happens within 2 years [5, 11, 12]. Nevertheless, studies from Sweden and the Netherlands show that BC is associated with higher SA as long as 5 years after diagnosis [13–15] and that some are granted DP up to 10 years after diagnosis [13, 15, 16]. Advanced cancer stage, [11–13, 17] chemotherapy, [15, 18] pre-diagnosis SA, [13–15, 19] comorbidity, [20] and several sociodemographic factors [12–15, 17, 19–30] were negatively associated with RTW, alternatively positively with SA/DP, depending on the outcome used. Thus, such variables need to be included in this type of studies. However, studies of SA, DP, and RTW vary greatly in terms of study design, outcomes, selection of included women, national health insurance systems, and female employment frequency, [5, 11, 16, 20–22, 31–33] the latter implying different health-selection effects on outcomes. Sweden has a high female employment rate, also in higher ages (> 50 years old) [34]. Thus, the healthy-selection effects on outcomes in Sweden are smaller which is an advantage when aiming at gaining knowledge on associations of BC with future SA/DP.

Most of the previous studies were based on short follow-ups, selected study populations, high drop-out, or only self-reported SA/DP, and lacked information on DP and pre-diagnosis SA/DP [20]. Although elevated levels of post-diagnostic anxiety and depression have been reported, [35, 36] detailed analyses of SA and DP due to mental diagnoses have seldom been conducted. Thus, knowledge is limited on pre- and post-diagnosis diagnosis-specific SA and DP in women with BC, in nationwide population-based studies; knowledge that is needed to better understand the situation for women diagnosed with BC, as a basis to identify potential risk factors for SA/DP as a basis for preventive measures. Moreover, in healthcare, among employers, insurance organizations, and patients, information regarding possible future SA following a BC diagnosis is asked for in order to take prevention measures and facilitate work accommodations [37]. Using information on disease-related and sociodemographic factors is one way to gain more such basic knowledge [38]. To the best of our knowledge, this is the first study to test a model for prediction of SA following a first BC diagnosis.

The aims were: (a) to explore the annual prevalence of SA and DP due to cancer, other somatic, and/or mental diagnoses during the 2 years before and the 3 years following a BC diagnosis, and (b) to predict risk of a new SA spell following a BC diagnosis.

## Methods

A population-based longitudinal cohort study was performed.

We included all the 3536 women in Sweden, aged 19–64 who were diagnosed with a first malignant neoplasm of breast (International Classification of Diseases 10th version (ICD-10) [39] code: C50) in 2010. Data were obtained from five nationwide registers as follows:

- The Board of Health and Welfare’s: Cancer Register (all BC cases 1958–2010, diagnosis date, type, T, N, and M classifications [40]), Patient Register (main diagnosis, dates of in- and specialized outpatient care 2008–2010), and Cause of Death Register (dates 2010–2013); - Statistics Sweden’s Longitudinal Integration Database for Health Insurance and Labor Market Studies (LISA) (age, educational level, marital status, family composition, birth country, occupational sector, geographical and type of living area in December 2009, emigration 2010–2012, not living in Sweden 2008 or 2009);

- National Social Insurance Agency’s Micro-data for Analyses of Social Insurance (MiDAS) (SA and DP benefits 2008–2013: dates, full- or part-time, main diagnosis).Data were linked at individual level using the ten-digit personal identity numbers assigned to all residents in Sweden [41].

### SA and DP public benefit schemes in Sweden

All people in Sweden ≥16 years, with an income from work or unemployment benefits, with reduced work capacity due to disease or injury can be granted SA benefit from the Social Insurance Agency [42]. The employers usually provide reimbursement for the first 14 days of a SA spell, which is why we do not have information on SA spells ≤14 days. From day 8, a medical certificate issued by the treating physician is required. SA spells can go on for long periods, even years. All residents aged 19–64, irrespective of labour market status, can be granted DP if having long-term or permanent work incapacity due to disease or injury. People aged 19–29 can be granted temporary DP. In those ages DP can also be granted if, due to disease or injury, needing more time to complete elementary or secondary school. SA and DP can be granted for full-time (100%) or part-time (25, 50, or 75%) of ordinary working hours, that is, people can be on partial SA and DP at the same time. SA benefits cover 80% and DP 64% of lost income, up to a certain level.

### Measures

We investigated two types of outcomes; DP and SA (for spells > 14 days). SA and DP days were transformed into net days; e.g., 2 days on half-time SA or DP were counted as one net day. SA and DP diagnoses were coded by the certifying physician who assessed the patient’s condition and work capacity. Diagnoses were for some of the analyses classified into four categories: 1): BC (ICD-10: C50), BC-related diagnoses (Z80, Z85, N61-N63), and other cancer (C00-D48), 2): mental diagnoses (F00-F99, Z73), 3): other diagnoses (all remaining ICD codes), and 4): missing information. The outcome in the predictive model was defined as starting a new SA spell > 14 days due to one of the following SA diagnoses (C00-D48, Z80, Z85, N61-N63, F00-F99, or Z73) during the time-window of 14 days before to 29 days after the BC diagnosis. This time window was based on the frequencies of start of new SA spells in the full cohort, in relation to diagnosis date (T_0_). For some women there was a delay before the diagnosis was included in the Cancer Register (even if the women were informed) and for others, treatment did not start until weeks later. The reason for including “diagnoses related to BC” and “other cancer diagnoses” in the predictive model was that sometimes a broader category of cancer diagnoses is given in the medical certificate [43]. Mental diagnoses were also included in the predictive model as a cancer diagnosis might lead to anxiety or depression [44, 45].

The included sociodemographic, disease-related, and comorbidity covariates (listed in Table 1) were selected for the predictive model based on previous findings regarding factors influencing SA and RTW [13, 14, 17, 20, 23, 25, 26, 29]. Missing information on educational level was coded as elementary school. Cancer-stage groups were assigned using the TNM Classification of Malignant Tumours [40] and categorized as: T0N0M0 + stage 0 + I, stage II, stage III + IV, and missing all TNM (with no T, N, or M information), respectively. When T, N, or M information was missing in one or two of the categories or classified as ‘X’ (assessment not possible), the value was set to 0. If more than one tumour was registered, with different diagnosis dates in 2010, the most advanced tumour was selected. The main ICD-10 diagnoses for healthcare were coded by the treating physicians. Healthcare due to uncomplicated delivery (O80) or not related to morbidity (e.g., screening) was excluded.

**Table 1:** Characteristics of the study cohort and the sub-cohort for modelling

*Covariates*	*The whole cohort*		*The cohort used for modelling*^*c*^	
	*Number*	*(%)*	*Number*	*(%)*
All	3536	100	2954	100
Age group				
18–35	122	3.5	111	3.8
36–45	665	18.8	610	20.7
46–50	583	16.5	515	17.4
51–55	630	17.8	525	17.8
56–60	844	23.9	664	22.5
61–63	692	19.6	529	17.9
Country of birth				
Sweden	2950	83.4	2509	84.9
Other country	586	16.6	445	15.1
Educational level				
Elementary school (≤9 years)	551	15.6	378	12.8
High school (10–12)	1549	43.8	1265	42.8
College/University (> 12)	1436	40.6	1311	44.4
Geographic living area				
North	425	12.0	344	11.7
Middle	486	13.7	403	13.6
Stockholm	823	23.3	710	24.0
West	934	26.4	767	26.0
South	868	24.6	730	24.7
Type of living area				
Larger cities	1376	38.9	1172	39.7
Medium cities	1252	35.4	1051	35.6
More rural areas	908	25.7	731	24.8
Family composition				
Married/cohab., no child at home	1042	29.5	852	28.8
Married/cohab., child at home	1235	34.9	1129	38.2
Single, no child at home	885	25.0	656	22.2
Single, child at home	374	10.6	317	10.7
Marital status				
Unmarried, divorced, widow	1523	43.1	1228	41.6
Married, registered partnership	2013	56.9	1726	58.4
Occupational sector				
Not in paid work/no information	810	22.9	379	12.8
Public	1409	39.9	1330	45.0
Private	1317	37.3	1245	42.2
Cancer stage				
Missing all T, N, M	25	0.7		
T0N0M0 + Stage 0 + I	2120	60.0	1799	60.9
Stage II	1162	32.9	990	33.5
Stage III + IV	229	6.5	165	5.6
Previous SA, net days^a,b^				
No previous SA	2870	81.2	2444	82.7
0.25–90	485	13.7	417	14.1
≥ 90	181	5.1	93	3.2
Previous SA, diagnoses^a^				
Mental diagnoses	161	4.6	124	4.2
Other diagnoses	540	15.3	412	14.0
Previous DP net days^a^				
0	2908	82.2	2771	93.8
0,25–365	180	5.1	153	5.2
≥ 365	448	12.7	30	1.0
Previous DP diagnoses^a^				
Mental diagnoses	181	5.1	47	1.6
Other diagnoses	466	13.2	137	4.6
Previous visits in specialized outpatient care^a^				
0	1549	43.8	1390	47.1
1–2 visits	1039	29.4	898	30.4
≥ 3 visits	948	26.8	666	22.6
Previous visits in outpatient care, diagnoses^a^				
Mental diagnoses	166	4.7	72	2.4
Other diagnoses	1825	51.6	1441	48.8
Previous inpatient care, days^a^				
0	3123	88.3	2709	91.7
1–14 days	354	10.0	235	8.0
≥ 14 days	59	1.7	10	0.3
Previous inpatient care, diagnoses^a^				
Mental diagnoses	32	0.9	11	0.4
Other diagnoses	391	11.1	238	8.1

The table included sociodemographic factors, cancer stage, and previous sickness absence (SA), disability pension (DP), and healthcare (n and %) for the cohort of all women in Sweden < 65 years with a first breast cancer diagnosis in 2010 as well as for those included in the logistic regression used to build a predictive model

^a^ Previous = in the period 730–15 days before the BC diagnosis date

^b^ The first 14 days of SA spells are not included

^c^ That is, those at risk for a new SA spell

### Statistical analyses

Different measures of SA and DP days were calculated.

In the in the period 730–15 days before the BC diagnosis date (T_0_): having had 0, > 0–90, or > 90 net SA days, having had 0, 0.25–365, or > 365 DP net days/year.

Per year, using the BC diagnosis date (T_0_) as reference: having had 0, > 0–30, > 30–90, > 90–180, or > 180 SA net days; in general, and by three SA diagnoses groups; having had any DP days/year, in general and by three DP diagnoses groups.

Moreover, the mean number of SA and of DP net days/year, respectively, were calculated for all women, using the BC diagnosis date (T_0_) as reference, for the 2 years before T_0_ and 3 years after T_0_ (Y_− 2_ to Y_+ 3_). This was done for all SA and DP as well as for the four SA/DP diagnostic categories mentioned above. The annual numbers and proportions of women with SA/DP due to the different diagnoses were also calculated. The denominator used in these calculations varied somewhat over the years due to the exclusion of women (due to turning 65 years, emigration, or death).

In the predictive model regarding risk of new SA related to time of diagnosis, 2954 women were included. For those analyses we excluded the 521 women (14.7%) already on SA or on DP for full-time or nearly full-time (75–100%) at T_0_. Additionally, 61 women were excluded due to lack of covariate information, or because of extreme values on some of the continuous variables, e.g., number of healthcare visits or inpatient days.

The risk of a new SA spell due to BC or related diagnoses, other cancer diagnoses, or mental diagnoses was modelled using multivariable logistic regression [46, 47] with a logistic model formulated as follows: log [P(Y_i_ = 1)/P(Y_i_ = 0)] = x_i_’β where Y_i_ denotes the SA status of individual *i*, and x_i_ is a vector of observed covariates. Natural cubic splines [48–50] were used to model potentially nonlinear effects of continuous covariates. We used two internal knots at the empirical quantiles 1/3 and 2/3. The five variables that were modelled using splines were: age and number of previous: SA days, DP days, outpatient healthcare visits, and inpatient days, respectively, in the two pre-diagnostic years. We developed two versions of the model: one without interactions; and another one with interactions between family composition-marital status, region-city size, previous SA days-outpatient visits, previous SA days-inpatient days. An optimal threshold *c* was selected, such that predicting *SA *whenever the fitted probability was above *c*, minimized the sum of false positive (FP) and false negative (FN) and maximized the proportion of correctly classified observations. Also, the receiver operating characteristic (ROC) was calculated. These values were also calculated using leave-one-out cross-validations.

## Results

Sociodemographic and diagnostic covariates are presented in Table 1 for the entire cohort (*N* = 3536) as well as for the group included in the modelling (*n* = 2954). About 40% of the women in both groups were ≥ 56 years. The compositions of women regarding distribution of characteristics in the two groups were fairly similar, except for percentage of women with no DP during the two pre-diagnostic years: 82% among all vs. 94% in the model, as expected due to the inclusion criteria in the modelling group. The majority had the earliest stages of BC. In the two pre-diagnosis years (Y_− 1_ and Y_− 2_), the majority had no SA days (81% vs. 83%), about half (56% vs. 53%) had at least one visit in specialized outpatient healthcare while few (12% vs. 8%) had at least one inpatient day. At BC diagnosis, 11.3% of the women were already on SA and 17.5% already on DP.

### Proportions of women with SA and/or DP

During the year after the BC diagnosis date (Y_+ 1_), 28% of the women had no SA > 14 days (Table 2), while 67% had SA due to cancer; nearly half of those (32%) for > 180 days. In the second year (Y_+ 2_), 35% of the women had at least some SA, regardless of SA diagnosis. For cancer SA diagnoses, the corresponding proportion was 25% during Y_+ 2_. During Y_+ 3_, the corresponding proportions were 25 and 12%, respectively. The proportions with SA due to mental diagnoses did not vary much between the studied years (3–4%). For SA due to other and missing diagnoses, the corresponding proportions were 8–11% all years, except for Y_+ 1_ when it was 6%. The proportion of women with DP ranged from 15 to 18% during all the five studied years. During Y_+ 1_, 15% of the women had neither SA nor DP. Before BC diagnosis, i.e., during Y_− 2_ and Y_− 1_, that proportion was 73%. During Y_+ 2_ and Y_+ 3_, the corresponding proportions were 52 and 62%, respectively.

**Table 2:** Number and percentages of women having different categories of number of sickness absence (SA) or disability pension (DP) net days per year, for the five studied years

SA/DP/ diagnoses	Categories of number of SA/DP days/year	Y_**−2**_ n (%)	Y_**−1**_ n (%)	Y_**+ 1**_ n (%)	Y_**+ 2**_ n (%)	Y_**+ 3**_ n (%)
		3522 (100)	3534 (100)	3536 (100)	3492 (100)	3191 (100)
**SA**^a^						
All	0	3115 (88.4)	3134 (88.7)	978 (27.7)	2282 (65.3)	2401 (75.2)
	> 0–30	198 (5.6)	206 (5.8)	485 (13.7)	422 (12.1)	324 (10.2)
	> 30–90	98 (2.8)	105 (3.0)	440 (12.4)	297 (8.5)	165 (5.2)
	> 90–180	55 (1.6)	50 (1.4)	366 (10.4)	213 (6.1)	133 (4.2)
	> 180	56 (1.6)	39 (1.1)	1267 (35.8)	278 (8.0)	168 (5.3)
Cancer^b^	0	3509 (99.6)	3506 (99.2)	1165 (32.9)	2610 (74.7)	2801 (87.8)
	> 0–30	≤8	19 (0.5)	486 (13.7)	306 (8.8)	124 (3.9)
	> 30–90	≤8	≤8	408 (11.5)	224 (6.4)	89 (2.8)
	> 90–180	≤8	≤8	337 (9.5)	151 (4.3)	75 (2.4)
	> 180	≤8	≤8	1140 (32.2)	201 (5.8)	102 (3.2)
Mental^c^	0	3423 (97.2)	3435 (97.2)	3430 (97.0)	3354 (96.0)	3074 (96.3)
	> 0–30	42 (1.2)	48 (1.4)	25 (0.7)	44 (1.3)	50 (1.6)
	> 30–90	22 (0.6)	30 (0.8)	22 (0.6)	43 (1.2)	21 (0.7)
	> 90–180	16 (0.5)	11 (0.3)	15 (0.4)	19 (0.5)	21 (0.7)
	> 180	19 (0.5)	10 (0.3)	44 (1.2)	32 (0.9)	25 (0.8)
Other^d^	0	3219 (91.4)	3246 (91.9)	3331 (94.2)	3208 (91.9)	2848 (89.3)
	> 0–30	158 (4.5)	153 (4.3)	85 (2.4)	151 (4.3)	205 (6.4)
	> 30–90	74 (2.1)	73 (2.1)	29 (0.8)	62 (1.8)	63 (2.0)
	> 90–180	37 (1.1)	36 (1.0)	16 (0.5)	30 (0.9)	35 (1.1)
	> 180	34 (1.0)	26 (0.7)	75 (2.1)	41 (1.2)	40 (1.3)
**DP**						
All	> 0	613 (17.4)	619 (17.5)	606 (17.1)	553 (15.8)	492 (15.4)
Cancer	> 0	≤8	≤8	12 (0.3)	13 (0.4)	21 (0.7)
Mental	> 0	171 (4.9)	170 (4.8)	160 (4.5)	148 (4.2)	135 (4.2)
Other^b^	> 0	445 (12.6)	449 (12.7)	436 (12.3)	393 (11.3)	338 (10.6)
**No SA/DP**	0	2578 (73.2)	2579 (73.0)	543 (15.4)	1812 (51.9)	1990 (62.4)
**Not included**^e^		14	≤8	0	44	345
Reasons for not being included the specific year						
> 65 years of age						248
Death ≤65 year of age					43	91
Not living in Sweden and ≤ 65 years		14	≤8		≤8	≤8

Included in the table were all women in Sweden < 65 years with a first breast cancer diagnosis in 2010 (*N* = 3536), during the 2 years before and 3 years after the breast cancer diagnosis date, presented for all SA/DP as well as by three categories of SA/DP diagnoses. Also, the number of women not included in the respective year are presented by reason for not being included

^a^ The first 14 days of SA spells are excluded

^b^ ICD-codes: C00-D48, Z80, Z85, N61-N63

^c^ ICD-codes: F00-F99, Z73

^d^ In the group” Other diagnoses”, also SA/DP with missing information on diagnosis were included

^e^ Women who turned 65, died, or emigrated were included up to and including the year of the event

### Mean SA and DP days/year

**Fig. 1:**
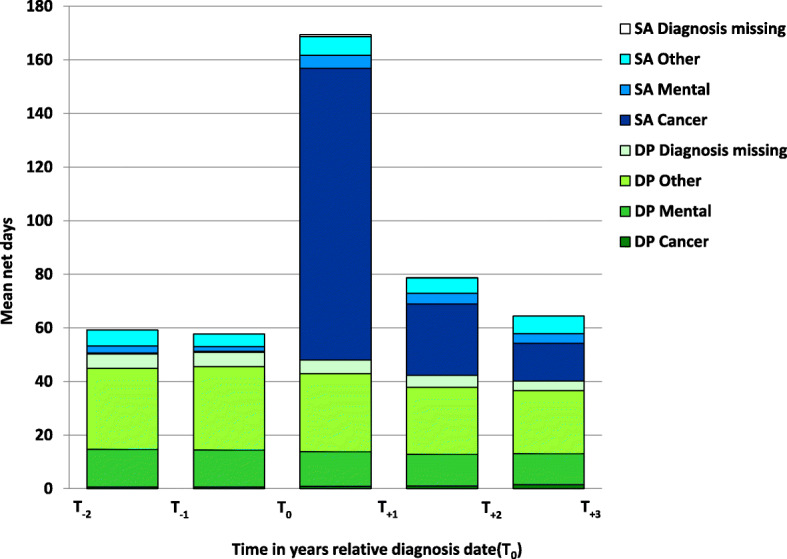
Mean annual number of sickness absence (SA) and disability pension (DP) net days by SA and DP diagnosis. Included in the figure: all women in Sweden < 65 years, with a first breast cancer diagnosis in 2010 (*N* = 3536), in the 2 years before and 3 years after date of diagnosis (T_0_), respectively. Included in the denominator each year: women < 65, alive, and living in Sweden

During Y_+ 1_, the mean number of SA days irrespective of SA diagnosis, among all the women in the cohort was 121.3. This was significantly higher than the numbers before diagnosis; (6.7 in Y_− 1_ and 9.0 in Y_− 2_) (Fig. 1). During Y_+ 1_, 108.8 of these SA days were due to cancer. That number was lower already in Y_+ 2_, i.e., 26.6 days. In Y_+ 3_, it was 14.0 days. Mean number of DP days/year was about 50 before BC diagnosis. Due to that some of the older women who already had DP in the year before T_0_ became 65 years of age, that number of DP days decreased to about 40 days/year in Y_+ 2_ and Y_+ 3_. Even in Y_+ 1_, the mean numbers of SA/DP days for the whole cohort was below less than half of the year.

### New SA spell

For the 3015 women who did not have an ongoing SA nor DP of the extent of 75–100% at the time of BC diagnoses (T_0_), Table 3 shows the numbers and percentages of women who had a first new SA spell in relation to T_0_. The same is shown for three specific SA diagnostic groups, i.e., cancer, mental diagnoses, and the other diagnoses (including missing), respectively. Of these women, 51% had a first new SA spell in relation to T_0_, that is, the period that was studied in the predictive modelling. Of these SA spells, 95% were due to cancer. In the following 30-day period, i.e., from 30 to 59 days after T_0_, another 20% had a first SA spell, of which 96% were with cancer. In the period from T_0_ until end of follow-up 3 years later, a little less than one fifth of the women (18%) had no new SA spells. Nine women were granted DP during that period. That is, about 80% of those at risk of a new SA spell following BC diagnosis, had such a spell in the first year (Y_+ 1_), and the majority of them (70%) in the first 3 months.

**Table 3:** Number and percentages of women with a new sickness absence (SA) spell (> 14 days)

Days relative to T_0_	Number (% of all women, column %)^a^	Cancer^c^ (row%)	Mental (row%)	Other diagnoses (row%)
No new SA spell before T_0_ to end of follow-up^c,^	536 (17.8)	–	–	–
14 days before T_0_ to 29 days after T_0_	1535 (50.9)	95	3	2
30–59 days after T_0_	599 (19.9)	96	1	3
60–89 days after T_0_	152 (5.0)	93	3	4
90–119 days after T_0_	78 (2.6)	92	1	6
120–179 days after T_0_	48 (1.6)	90	0	10
180–364 days after T_0_	19 (0.6)	68	0	32
365–729 days after T_0_	21 (0.7)	57	14	29
≥730 days after T_0_	27 (0.9)	18	15	67

The included individuals were 3015 women < 65 years related to date of a first breast cancer diagnosis in 2010 (T0), during the following 3 years; all SA and diagnosis-specific SA (cancer, mental, or others)

^a^ Women already (nearly) full-time (75–100%) SA or disability pension (DP) at T_0_ were not included

^b^ ICD-codes: C00-D48, Z80, Z85, N61-N63

^c^ Nine of these women were granted DP during follow-up

### Predictive model

**Fig. 2:**
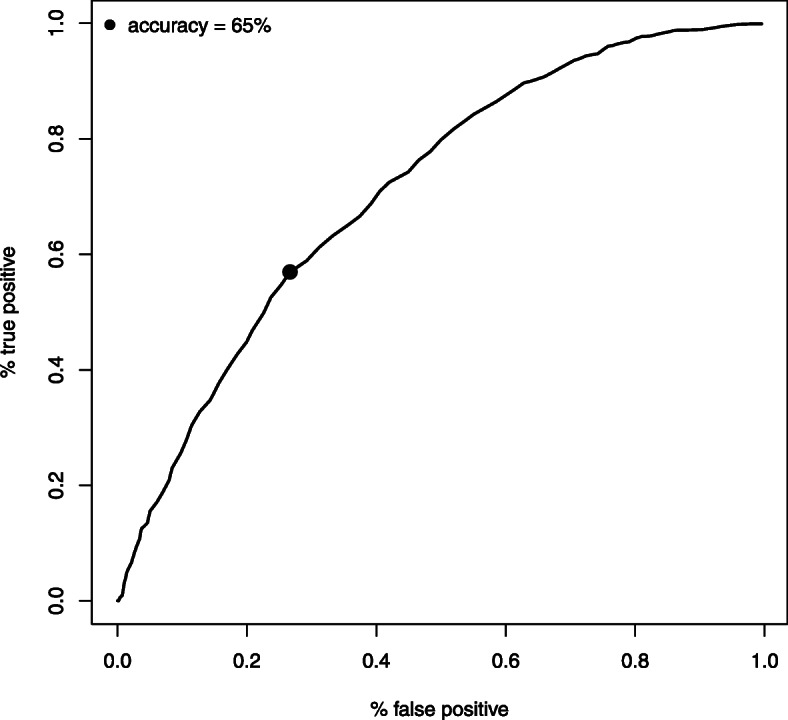
The receiver operating characteristic (ROC) curve. Proportion of false positive/negative at different values of *c* in the cohort of women in Sweden < 65 years with a first breast cancer diagnosis in 2010. The dot corresponds to the optimal choice of threshold *c*

The model without interactions had similar cross-validated ROC-AUC as the model with interactions (results not shown). We, therefore, choose to report results for the simpler model without interactions for the sake of parsimony. Out of the variables (see Table 1) included in the multivariable logistic regression model for the risk of having a new SA spell > 14 days (due to BC or related diagnoses, other cancer diagnoses, or mental diagnoses) in connection with a first BC diagnosis (T_0_), the following variables were statistically significant (*p* < 0.05): occupational sector, living area, age, cancer stage, educational level, and number of previous net SA days (listed according to predictive strength from high to low). In Fig. 2, the receiver operating characteristic (*ROC*) curve is illustrated. The dot indicates the coordinates (FP, FN) corresponding to the selected value of threshold *c*. The predictive model could correctly predict 35% out of 2954 women, given the optimal threshold 0.56. Results are summarized in Table 4 where the area under the curve (AUC) [51, 52] is also reported.

**Table 4:** Performance measures of model without interactions, after leave-one-out cross-validations

	Values
% False positive	13
% False negative	21
% Correct	35
Sensitivity	0.57
Specificity	0.73
*Area under the curve (AUC)*	0.71

The values included in the table were: proportion of false positive (FP) and false negative (FN), proportion of correctly classified observations, and area under the curve (AUC) in the analyses of women in Sweden < 65 years with a first breast cancer diagnosis in 2010 (*N* = 2954)

1 Women not at risk of a new SA spell at date of breast cancer diagnosis were not included (that is, those already on (nearly) full-time SA or DP (75–100%)

## Discussion

In this longitudinal population-based cohort study of all women in Sweden aged 19–64 years with a first BC diagnosis in 2010, the proportion with SA > 14 days increased substantially in the post-diagnostic 12 months. Nevertheless, most women were not on SA for extensive times during the first year after diagnosis date (e.g., > 180 net days) and about 17% were already on SA/DP due to other diagnoses when diagnosed with BC. In the third year after BC diagnosis date (Y_+ 3_), the mean number of SA and DP days was low and 25% of the women had no SA at all. The variation in proportion of women with DP was minor during the 5 years being studied. The elevated proportion of women on SA and the higher number of SA days after BC diagnosis, compared to the years before BC diagnosis, was in SA due to cancer throughout follow-up, e.g., not due to mental diagnoses. Of the 3015 women not already on SA/DP at time of BC diagnosis, 51% had a new SA spell and for 98% of them the SA was due to a cancer diagnosis, cancer-related diagnosis, or mental diagnosis. Using a predictive model including disease-related and socio-demographic factors, 67% of the women could be correctly classified into having or not having a new SA spell, indicating that it would be possible to predict risk of future SA among working-aged women diagnosed with BC.

### Strengths and limitations

The data from the entire population of Sweden provided us with a rare opportunity to study SA and DP in connection with BC diagnosis in all women of working age. Other strengths are the longitudinal cohort design, that all women fulfilling the inclusion criteria of a first BC diagnosis in a whole country could be included, not a sample; also that extensive microdata on morbidity and sociodemographic variables from several high-quality registers could be linked at individual level [41, 53, 54]; and that data were not self-reported, avoiding recall bias. The large cohort also allowed sub-group analyses of specific SA/DP diagnoses, not only the BC diagnosis. The latter circumstance makes the results and model more useful in practice. High female employment frequency, complete coverage of the public SA/DP insurances, and no dropouts make the internal validity of the study very strong. A further strength is that we were able to exclude women when they during follow-up, due to death, turning 65, or emigration no longer were at risk of the outcome SA/DP. Findings can be generalized to women with BC in countries with comparable employment frequencies and coverage of SA/DP benefits. Another strength was the use of several different measures of SA and DP, which provided a wide picture of the complex data that SA and DP data provide, e.g., regarding skewed distribution, regarding occurrence of spells and durations of spells and time between spells, different diagnoses and seriousness [55–57].

Regarding the predictive model, we found that the specified interactions did not improve the predictive performance of the model. While it is possible that other interactions between included variables exists, we expect limited gains in terms of predictive power from the inclusion of more interactions, as well as a risk for overfitting. The aim here was to explore if predictions could be possible, which we found. Future studies need to further develop such analyses, both regarding this outcome and others, e.g., durations of SA spells.

The validity of SA and DP diagnoses is sometimes discussed but seldom investigated. To the best of our knowledge, there is only one such study, regarding SA diagnoses, [43] and that study found the validity to be acceptable. The validity of DP diagnoses is likely to be even higher, since DP is only granted after a long process of medical evaluation [42]. However, the stigma associated with mental diagnoses [58] might imply an underestimation of SA/DP due to such diagnoses. Other limitations of our study are that we had no information on SA spells ≤14 days and only information about the first and main SA diagnosis of a SA spell. Further, we did not have information on cancer treatment, which is a factor that might attenuate the association between cancer stage and SA [14, 15]. Previous studies have, e.g., shown that chemotherapy is associated with higher levels of SA/DP [15, 18].

### Discussion of results

We were able to replicate previous findings that the prevalence of SA was considerably higher in the year post-BC diagnosis than before diagnosis, using a population-based nationwide cohort [13, 14]. In a previous Swedish population-based study of all women aged 20–65 with a first BC diagnosis in 2005, [13] almost the same proportion of women as here presented (71% vs. 72%) had some SA during the first 12 months following BC diagnosis. In the cohort from 2005, however, a larger proportion of women, i.e., 19%, had SA already in the year before BC diagnosis, compared to 11% in our 2010 cohort. Also, proportions on DP were slightly higher in the pre-diagnostic year (Y_− 1_) in the 2005 cohort compared to the current cohort, 20.6% vs. 17.5%. In the following 2 years, the decline in SA was somewhat faster in our 2010 cohort. This difference might imply an impact of the stricter Swedish SA/DP regulations implemented in 2008 [59, 60]. However, the hypothesis should be studied further using other study designs.

The higher prevalence of SA after BC diagnosis, compared to the pre-diagnostic period, was due to cancer only, not due to mental nor to other somatic diagnoses in neither our cohort nor the 2005 cohort used in the previous Swedish study from our group, [13] i.e., SA due to mental diagnoses did not increase after the BC diagnosis. This is noteworthy, as studies have reported higher risks of anxiety and depression after BC diagnosis [35, 36]. One possible explanation is that mental disorders in women with BC are not recognized by sickness certifying physicians [61]. Another explanation is that such mental disorders did not reduce work capacity to such a high level that SA was required or that the women, if needed, soon had adequate treatment for mental disorders – more knowledge is needed on this. It is also possible that mental diagnoses was stated as a secondary SA diagnosis or becoming a main SA diagnosis later during the SA spell [62] and thus not captured by our data.

Regarding the predictive model, our ROC.AUC of 0.71 is considered below recommended levels for clinical use [38, 63]. Nevertheless, the results from the model are promising regarding the possibility to further develop a predictive model. It also can be used to inform patients, healthcare staff, and other stakeholders that all women diagnosed with BC do not require SA very soon.

Our results highlight the importance of communicating to women with BC as well as to employers of the fact that most women with BC return to work rather quickly, in order to promote optimal work adjustments as soon as possible, especially for groups with an elevated risk of SA/DP.

## Conclusions

In this population-based prospective cohort study we found that although BC and BC treatment can have large impacts on work capacity, not all women diagnosed with BC had extensive SA/DP even in the first 12 months after diagnosis. Moreover, it is possible to give a good prediction of which women with BC who are at high risk of new SA. Our predictive model should be further developed to assist the RTW measures for people with cancer diagnosis in the future.

## Abbreviations

BC: Breast cancer
SA: Sickness absence
DP: Disability pension
RTW: Return to work
ICD-10: International Classification of Diseases 10th version
LISA: Longitudinal Integration Database for Health Insurance and Labor Market Studies
MiDAS: Micro-data for Analyses of Social Insurance
FP: False positive
FN: False negative
ROC: Receiver operating characteristic
AUC: Area under the curve
